# Discontinuation of tyrosine kinase inhibitors based on *BCR-ABL1* monitoring by digital droplet PCR in pediatric chronic myeloid leukemia

**DOI:** 10.3389/fped.2022.928136

**Published:** 2022-07-27

**Authors:** Yeojae Kim, Seongkoo Kim, Jong Mi Lee, Ari Ahn, Jae Won Yoo, Jae Wook Lee, Bin Cho, Nack-Gyun Chung, Yonggoo Kim, Myungshin Kim

**Affiliations:** ^1^Catholic Genetic Laboratory Center, College of Medicine, Seoul St. Mary's Hospital, The Catholic University of Korea, Seoul, South Korea; ^2^Department of Biomedicine and Health Sciences, Graduate School, The Catholic University of Korea, Seoul, South Korea; ^3^Division of Pediatric Hematology/Oncology, Department of Pediatrics, The Catholic University of Korea, Seoul, South Korea; ^4^Department of Laboratory Medicine, College of Medicine, The Catholic University of Korea, Seoul, South Korea

**Keywords:** chronic myeloid leukemia, tyrosine kinase inhibitor, *BCR-ABL1*, digital droplet PCR, molecular response, discontinuation, children

## Abstract

Lifelong treatment of pediatric chronic myeloid leukemia (CML) patients with tyrosine kinase inhibitors (TKIs) can affect their growth and development. For these reasons, clinical trials have explored the feasibility of TKI discontinuation in children with a sufficient TKI response. We evaluated the analytical performance of digital droplet PCR (ddPCR) to quantify *BCR-ABL1* and compared the results with reverse transcription quantitative polymerase chain reaction (RT-qPCR). We further investigated whether ddPCR could be used to determine TKI discontinuation in a clinical setting. Performance of ddPCR was evaluated using standard materials for *BCR-ABL1*, and a total of 197 clinical samples from 45 pediatric CML patients was included for comparison with RT-qPCR. ddPCR showed excellent analytical sensitivity with 0.001% international scale (IS) and linearity with *R*^2^ > 0.99 in log scale. *BCR-ABL1* % IS results correlated well with those of RT-qPCR (*R*^2^ = 0.9435), however, they showed a moderate strength for agreement with a Cohen's kappa of 0.41 due to higher sensitivity of ddPCR. Among 45 pediatric CML patients, 42 were treated with first-line TKIs including imatinib (*n* = 27, 64%) and dasatinib (*n* = 12, 29%), and three patients that were started with imatinib were switched to dasatinib. When we evaluated whether follow-up samples fulfilled *ABL1* copies ≥ 10,000 required for deep molecular response (DMR), all samples were acceptable by ddPCR, whereas 18% by RT-qPCR did not reached acceptable *ABL1* copies. Moreover, 52 and 13% reached *ABL1* copies ≥ 32,000 required for MR4.5 by ddPCR and RT-qPCR, respectively. Seven patients discontinued TKI and the median TKI treatment duration was 73 months prior to discontinuation. Prior to discontinuation, the median duration of sustained undetected *BCR-ABL1* was 60 months. Two patients experienced loss of major MR (MMR) during follow-up and restarted dasatinib 5 months after discontinuation. They achieved MMR again and maintained better than DMR afterward. Results from those patients demonstrated that RT-qPCR did not match the need for adequate *ABL1* copies for MR4.5 while majority of ddPCR could. Therefore, ddPCR was technically more acceptable to decide and monitor pediatric CML patients before and after TKI discontinuation.

## Introduction

Tyrosine kinase inhibitors (TKIs) are a cornerstone of successful clinical management of chronic myeloid leukemia (CML) patients. Since the introduction of TKIs as a treatment strategy in CML, prognoses have improved considerably not only in adult patients, but also in children (1, 2). Imatinib and the second-generation TKIs dasatinib and nilotinib were recently approved as a first-line treatment in children, expanding treatment options and allowing allogeneic stem cell transplantation (allo-SCT) as a third-line treatment (3). However, lifelong treatment of children treated with TKI can affect their growth and development (4, 5). For these reasons, clinical trials have explored the feasibility of TKI discontinuation in children with a sufficient TKI response (6). International guidelines have proposed discontinuation of TKI in selected adult patients with a deep and sustained molecular response (7, 8). Careful monitoring of the molecular response (MR) of each patient using highly sensitive methods with at least *BCR-ABL1* MR4.5 and ≤ 0.0032% on the international scale (IS) is essential to select patients (9).

Reverse transcription-quantitative PCR (RT-qPCR) has been widely used for evaluating patient response to TKIs (10, 11). However, RT-qPCR shows limited sensitivity and precise monitoring of residual disease, specifically in patients who maintain a stable deep molecular response (DMR) under TKI treatment (12). Digital droplet PCR (ddPCR) is a method for performing digital PCR based on water-oil emulsion droplet technology. The fractionation of an analyte into 20,000 droplets and PCR amplification in each individual droplet enables end point measurement and absolute quantification of a target sequence. The use of ddPCR for quantitative *BCR-ABL1* monitoring was not only reliably detected in DMRs relevant to guiding treatment, but also helped to better identify patients prone to relapse after stopping TKI treatment (13, 14).

Although pediatric and adult CML share common molecular pathogenesis and hematologic characteristics, CML studies in children and adolescents are different from those in adults (3, 15). Data are not yet sufficient with regard to long-term efficiency and safety specific to pediatric patients but also with regard to TKI discontinuation. A recent study indicated that imatinib could be discontinued without an impact on outcomes in patients younger than 18 years at diagnosis of CML who responded well to treatment (no failure, no suboptimal response, and no switch to another TKI) and achieved sustained MR4 for at least 2 years with imatinib (11). However, TKI discontinuation in pediatric CML still relies on experience in adults.

In this study, we quantified *BCR-ABL1* using RT-qPCR and ddPCR in pediatric CML patients treated with TKI. Subsequently, we evaluated the performance of ddPCR using reference materials and available clinical samples. Finally, we aimed to demonstrate the feasibility of ddPCR to determine TKI discontinuation and to monitor steady DMRs for pediatric CML patients by comparing the results with those of RT-qPCR.

## Materials and methods

### Patients and samples

We reviewed medical records of 45 pediatric CML patients who were diagnosed with chronic myeloid leukemia and were treated in Seoul St. Mary's Hospital from November 2009 to March 2021. Standard diagnoses were established according to the WHO Classification of Tumors of Haematopoietic and Lymphoid Tissues based on bone marrow (BM) morphology and cytogenetic and molecular genetic analyses (16). Among them, 42 patients were prescribed TKI. The median patient age was 12 years (range, 2–17 years), and 23 patients (55%) were men. The clinical and laboratory characteristics are summarized in Table 1. Among the consecutive cohorts, 31 patients were in the chronic phase (74%), 6 in the accelerated phase (14%), and 5 in the blast phase (12%) at diagnosis. Patients were treated with first-line TKIs including imatinib (*n* = 30, 71%) and dasatinib (*n* = 12, 29%). Three patients were started with imatinib and switched to dasatinib. The median duration of follow up was 48 months (range, 6–138) for TKI treatment. Molecular responses (MRs) were monitored using RT-qPCR analysis at 3-month intervals and at 3- or 6-month intervals after major molecular response (MMR) was achieved. MR1, MR2, MR3 (MMR), MR4 (DMR), MR4.5, and MR5 are defined as the transcript ratio of *BCR-ABL1/ABL1* being 10% or less, 1% or less, 0.1% or less, 0.01% or less, 0.0032% or less, and 0.001% or less, respectively, obtained from peripheral blood leukocytes on an international scale (IS) (17). All patients were carefully reevaluated with regard to % *BCR-ABL1* by ddPCR prior to discontinuation. Seven patients met the criteria for and were discontinued from TKI. After discontinuation, *BCR-ABL1* was carefully monitored using both RT-qPCR and ddPCR.

**Table 1:** Patients characteristics.

**Variables**	***n*** = **42**
**Median Age, years (range)**	12 (2–17)
**Sex, *n* (%)**
Female	19 (45%)
Male	23 (55%)
**Disease stage at diagnosis, *n* (%)**
Chronic phase	31 (74%)
Accelerated phase	6 (14%)
Blast phase	5 (12%)
**Fusion gene, *n* (%)**
*b3a2*	29 (69%)
*b2a2*	7 (17%)
*e14a2*	1 (2%)
Not applicable	5 (12%)
**CBC at diagnosis, count (range)**
White blood cell (×10^9^/L)	292.1 (12.9–562.7)
Hemoglobin (g/dL)	8.8 (5.4–14.2)
Platelet (×10^9^/L)	459.5 (33.0–1,720.0)
**Splenomegaly, *n* (%)**	32 (76%)
**Initial TKI, *n* (%)**
Imatinib	30 (71%)
Dasatinib	12 (29%)
**Median duration of follow up, months (range)**	48 (6–138)

*n, numbers; CBC, complete blood count; TKI, tyrosine kinase inhibitor*.

### RT-qPCR and ddPCR

RT-qPCR was performed using 1 μg of total RNA with a Real-Q *BCR-ABL* Quantification Kit (BioSewoom, Seoul, Republic of Korea) using an Applied Biosystems® 7500 Fast Dx Real-Time PCR Instrument (Thermo Fisher Scientific Inc., Waltham, Massachusetts, US) according to the manufacturer's instructions. We analyzed *BCR-ABL1/ABL1* by ddPCR in addition to RT-qPCR after patients achieved MMR. In addition, 1,000 ng of total RNA was transcribed to cDNA by the Transcriptor First Strand cDNA Synthesis Kit (Roche Diagnostics GmbH, Manheim, Germany) using a GeneAmp® PCR System 9700 (Thermo Fisher Scientific Inc., Waltham, Massachusetts, US). PCR was performed with a QXDx™ *BCR-ABL* %IS Kit (Bio-Rad Laboratories, Hercules, California, US) using a QX200™ Droplet Digital PCR System according to the manufacturer's instructions. The PCR reaction mix generated approximately 20,000 droplets per well by a Droplet Automated Droplet Generator (Bio-Rad Laboratories, Hercules, California, US), and PCR was performed using an Arktik™ Thermal Cycler (Thermo Fisher Scientific Inc., Waltham, Massachusetts, US). Data were analyzed by the QX200 Droplet Reader and QuantaSoft™ Software version 1.7.4 (Bio-Rad Laboratories, Hercules, California, US). Results were calculated based on raw data using QXDx™_*BCR-ABL*_Reporter_v.1.02 (b168, Bio-Rad Laboratories, Hercules, California, US). The modification conversion factor value and minimum and maximum calibration qualities are supported by the manufacturer instrument. The absolute numbers of *BCR-ABL1* and *ABL1* copies were determined by Poisson statistics. Results were reported when the *ABL1* copies were at least 10,000 per well.

The analytical performance of ddPCR was evaluated using *BCR-ABL1* negative clinical samples and certified reference material (CRM) with four levels of *BCR-ABL1* mRNA ranging from 0.01 to 10% (10-fold), 1st WHO International Genetic Reference Panel for the quantitation of *BCR-ABL1* translocation (Product code 09/138, National Institute for Biological Standards and Control, Herts, United Kingdom) containing freeze-dried K562 cells (*BCR-ABL1* positive, *e14a2*, also known as *b3a2*), and HL60 cells (*BCR-ABL1* negative). Linearity was calculated using eight replicates of each concentration. Comparison between RT-qPCR and ddPCR was performed using data from 197 clinical samples.

### Statistics

Linearity of *BCR-ABL1/ABL1* (% IS) by ddPCR was performed using polynomial and linear regression. Wilcoxon signed rank test was used to compare *BCR-ABL1* and *ABL1* copy numbers by RT-qPCR and ddPCR. The consistency of *BCR-ABL1* detected vs. undetected and MRs between RT-qPCR and ddPCR were analyzed by Cohen's kappa and Chi-square test. Pearson's correlation analysis and linear regression were also performed to compare quantitative results, and a scatter plot was presented. All statistical analyses were performed using SPSS Statistics 20 (IBM, Armonk, NY, USA) and Microsoft Excel 2019 (Microsoft, Redmond, WA, USA) software.

## Results

### Analytical and clinical performance of ddPCR

**Figure 1:**
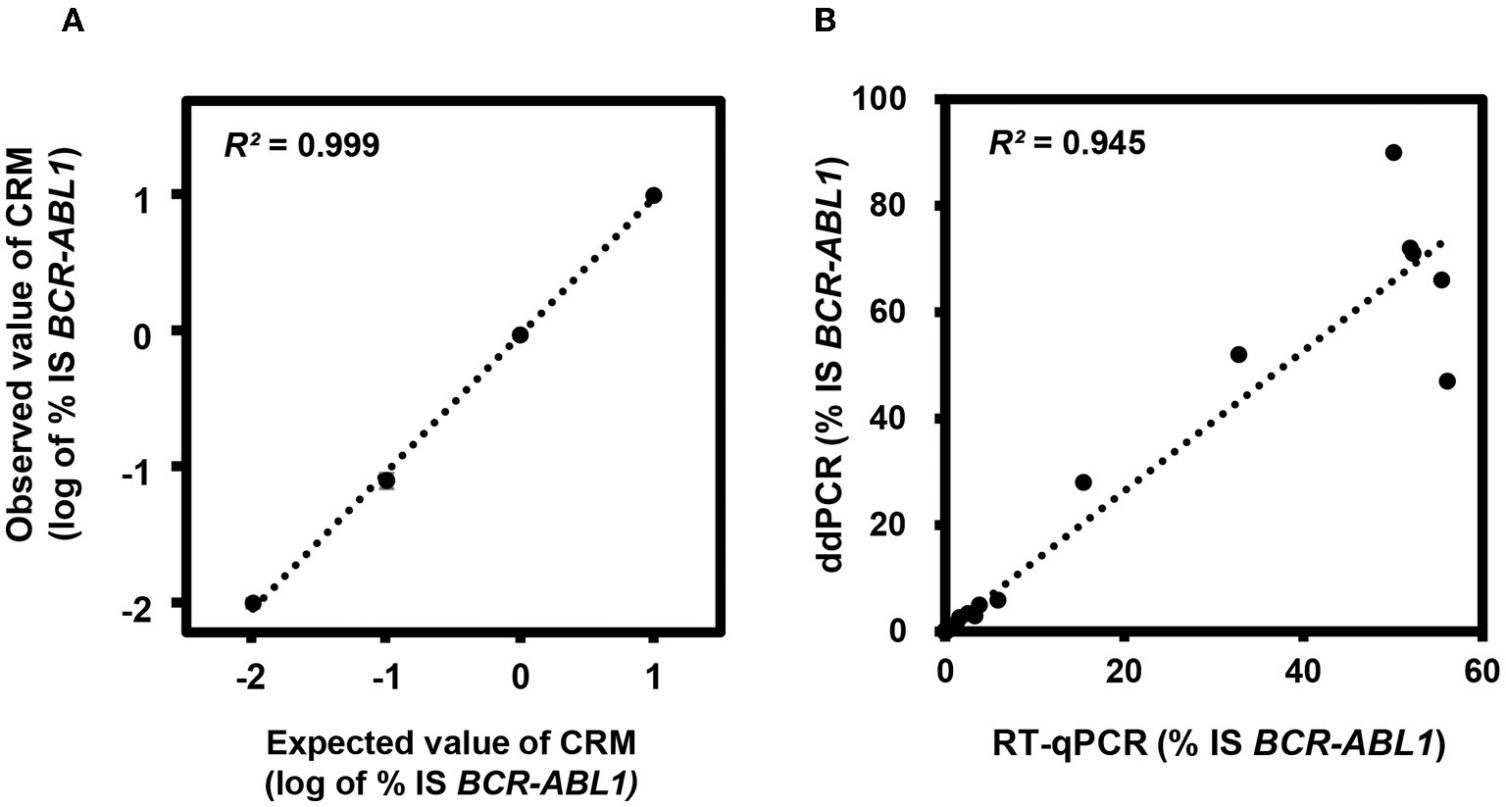
Analytical performance of digital droplet polymerase chain reaction (ddPCR) for *BCR-ABL1*. **(A)** Linearity analysis using certified reference materials (CRMs). **(B)** Correlation between reverse transcription quantitative PCR (RT-qPCR) and ddPCR in clinical samples. IS, international scale.

First, we evaluated the analytical performance using CRMs and found that ddPCR showed excellent sensitivity with 0.001% IS and linearity with *R*^2^ = 0.9988 in log scale (Figure 1A). The CV was 0% for 10% IS CRM, 0.05% for 1% IS CRM, 0.014% for 0.1% IS CRM, and 0.001% for 0.01% IS CRM. Then, we evaluated the clinical performance using real world samples with a wide range of *BCR-ABL1* (median 0.0044% IS, 0%-90%). Results from ddPCR correlated well with those from RT-qPCR (*R*^2^ = 0.945, *p* <0.001) (Figure 1B). Meanwhile, copy numbers of *BCR-ABL1* and *ABL1* were significantly low when measured by RT-qPCR (*p* <0.001 and <0.001, respectively).

### Clinical performance between RT-qPCR and ddPCR

**Figure 2:**
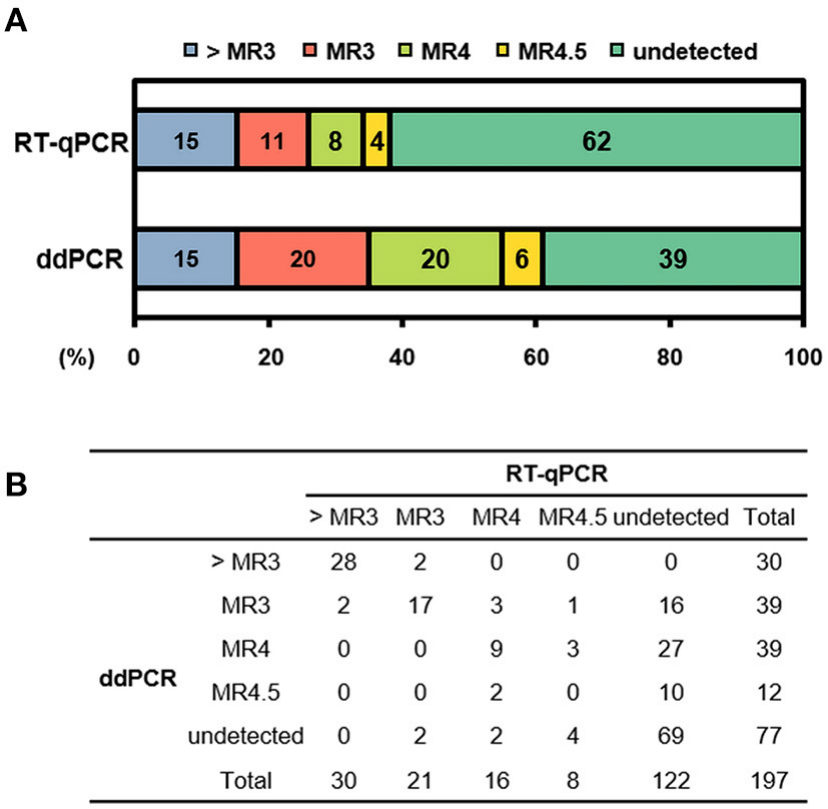
Distribution of molecular response (MR) according to detection method. **(A)** Stacked column chart of MR. The bars are color-coded at the MR level with percentage of sample numbers. **(B)** Distribution of number of samples in each MR. > MR3, not achieve MR3; RT-qPCR, reverse transcription quantitative polymerase chain reaction; ddPCR, digital droplet PCR.

In terms of the consistency of detected and undetected *BCR-ABL1*, 136 of 197 results (69%) showed consistent results by RT-qPCR and ddPCR. Sixty-seven samples (34%) were both-positive and 69 samples (35%) were both-undetected. We found inconsistency in 61 samples, revealing a moderate strength for agreement with a Cohen's kappa of 0.41. The majority was ddPCR-detected but RT-qPCR-undetected (*n* = 53, 87%), whereas only 8 samples (13%) were ddPCR-undetected but RT-qPCR detected. Because most inconsistent results were from patients after treatment, we compared the results according to MR. The comparison included all available samples for MR evaluation and showed a significant difference between the two methods (*p* <0.001 by χ^2^ test). We found inconsistent MR in 74 samples: 62 samples showed lower %IS by RT-qPCR, whereas only 12 samples did by ddPCR when comparing based on the cut-off for MR (Figure 2).

Next, we investigated the number of *ABL1* copies in samples showing better than DMR (MR4.0, MR4.5, and undetected, *n* = 89). When we evaluated whether those samples fulfilled *ABL1* copies ≥ 10,000 required for DMR, all samples were acceptable by ddPCR, whereas 18% (*n* = 16) by RT-qPCR did not reached acceptable *ABL1* copies. Among them, 46 and 12 samples (52 and 13%) reached *ABL1* copies ≥ 32,000 required for MR4.5 by ddPCR and RT-qPCR, respectively.

### *BCR-ABL1* before and after TKI discontinuation

**Figure 3:**
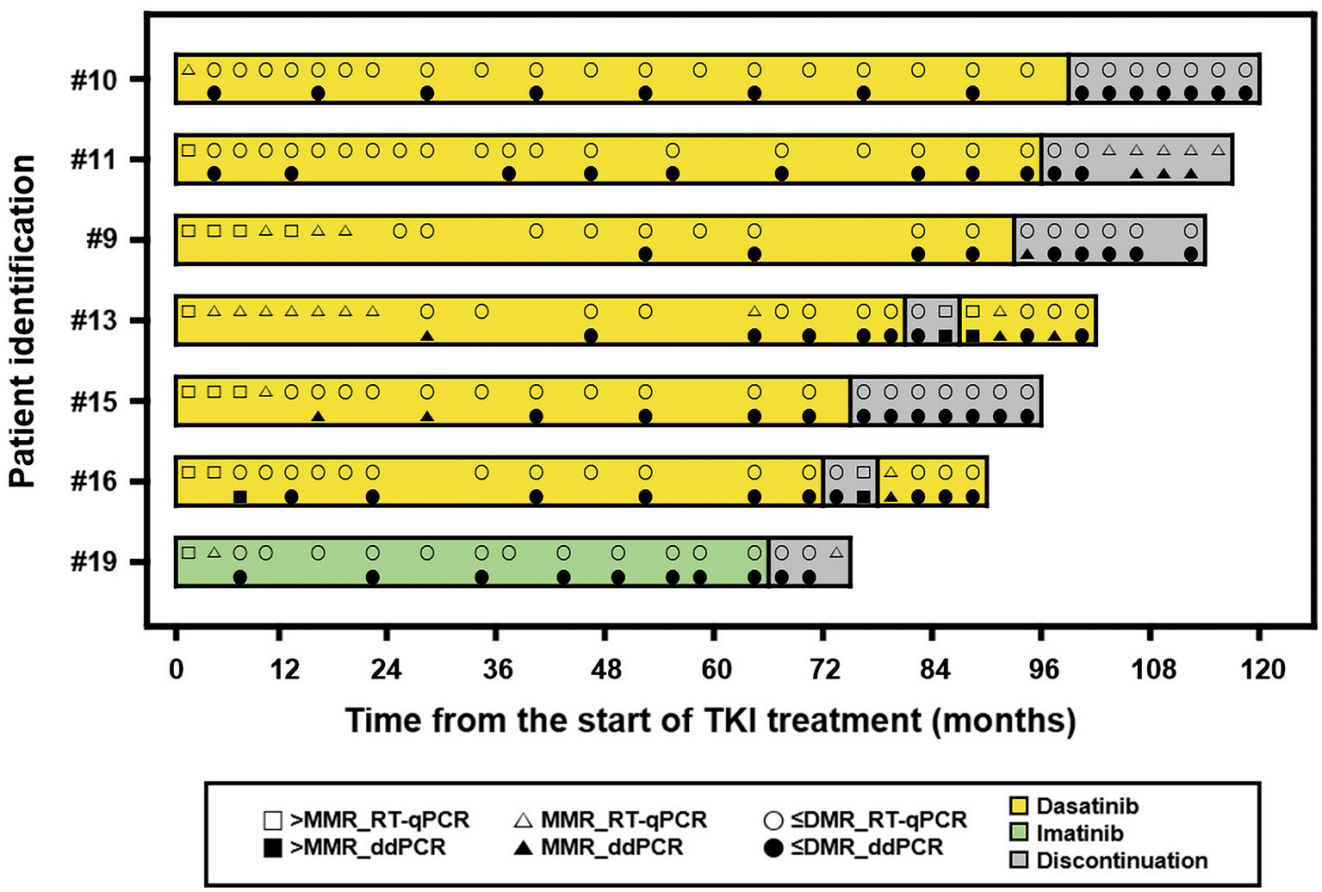
A swimmer plot showing the course of discontinuation of tyrosine kinase inhibitor (TKI). The band represents an individual patient. > MMR, not achieved major molecular response; RT-qPCR, reverse transcription quantitative polymerase chain reaction; ≤ DMR, achieved response better than deep molecular response including undetected; ddPCR, digital droplet PCR.

The achievement rate of MMR was 7% in the first 3 months, 31% at 6 months, and 50% at 12 months. Patients achieving DMR comprised 17% at 6 months, 29% at 12 months, and 40% at 18 months. The achievement rate of MR4.5 was 7% at 6 months, 24% at 12 months, and 36% at 18 months. Seven patients sustained undetected *BCR-ABL1* by both RT-qPCR and ddPCR for at least 2 years. Six were treated with dasatinib and the other with imatinib. The median TKI treatment duration was 73 months (range, 69–97) prior to discontinuation. The median age at TKI discontinuation was 20 years (range, 9–25). Prior to discontinuation, the median duration of sustained undetected *BCR-ABL1* was 60 months (range, 51–93), and the median number of successive *BCR-ABL1* measurements better than DMR was 9 (range, 8–15). None of the RT-qPCR results showed *ABL1* copies ≥ 32,000, while 55% of ddPCR results showed this level. TKI was discontinued with permission of patients and their parents. The *BCR-ABL1* level was monitored every 2 months after discontinuation. The course of each patient can be seen in Figure 3. Two patients experienced loss of MMR during follow-up and restarted dasatinib 5 months after discontinuation. They achieved MMR again and maintained better than DMR afterward.

**Figure 4:**
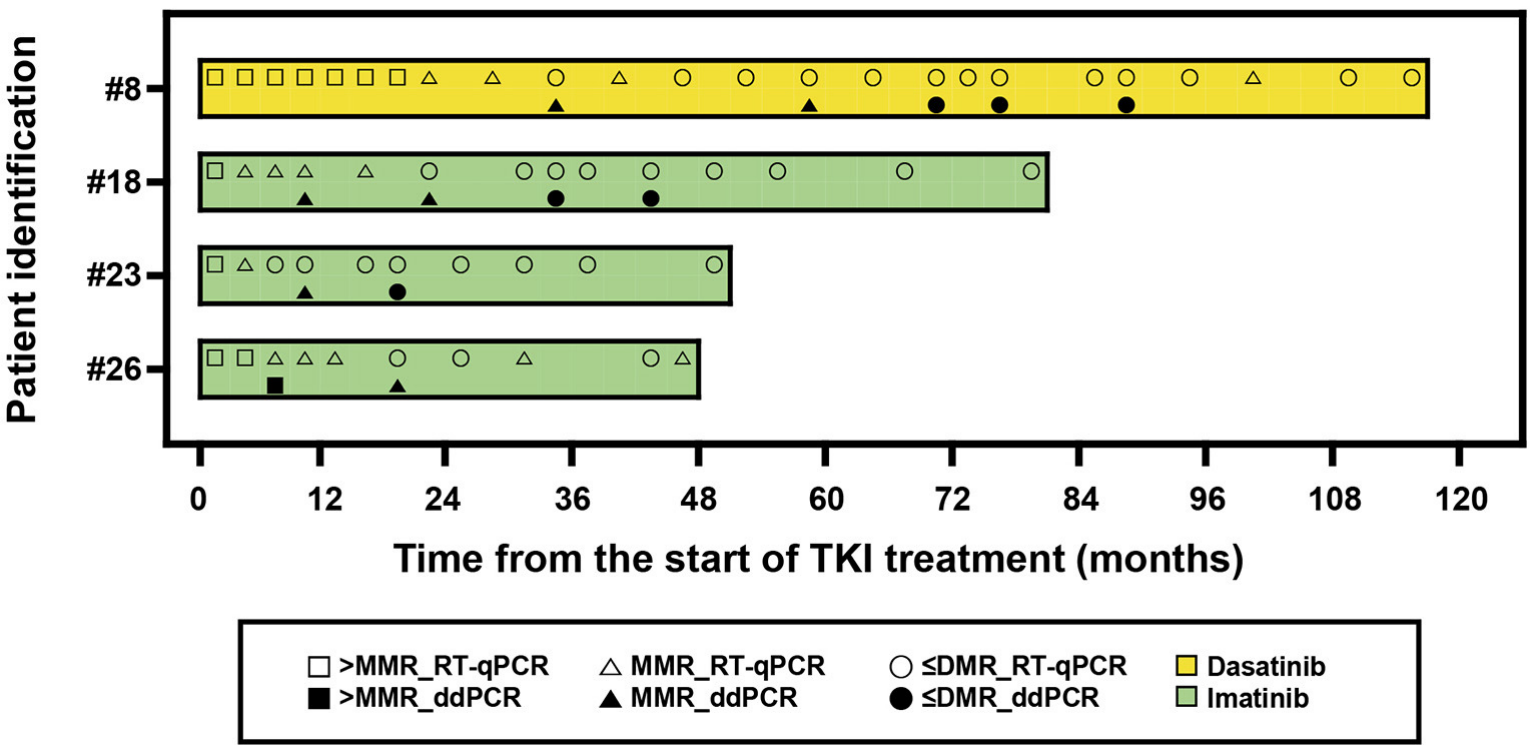
A swimmer plot showing the molecular response of patients treated by tyrosine kinase inhibitors (TKIs) more than 24 months. The band represents an individual patient. > MMR, not achieved major molecular response; ≤ DMR, achieved response better than deep molecular response including undetected; RT-qPCR, reverse transcription quantitative polymerase chain reaction; ddPCR, digital droplet PCR.

We further analyzed four patients who maintained TKI treatment more than 24 months and compared *BCR-ABL1* results (Figure 4). The median TKI treatment duration was 66 months (range, 48–117). Among 13 results, six showed different MR between RT-qPCR and ddPCR. ddPCR revealed worse MR than RT-qPCR. None of the RT-qPCR results showed *ABL1* copies ≥ 32,000, while all ddPCR results showed this level.

## Discussion

Accurate measurement of *BCR-ABL1* is essential for optimal management of CML patients treated with TKIs. This method has gained significance since discontinuation of TKI was considered in patients with sustained DMR. The low levels of *BCR-ABL1* typically achieved during TKI treatment demand a sensitive monitoring assay for reliable detection and quantitation (18). RT-qPCR has proven to be an effective and clinically validated laboratory method to quantify *BCR-ABL1* level and was the most widely used to assess the response of TKI. In recent years, ddPCR has appeared as a more sensitive and accurate tool for monitoring CML patients. Studies suggest ddPCR as an alternative approach to RT-qPCR to improve the recognition of stable DMR. Although ddPCR is not yet widely applied in clinical laboratories, it will be most useful for selection of patients for treatment discontinuation and for prediction for treatment-free remission. In line with previous studies, ddPCR showed excellent analytical performance with 0.001% IS sensitivity and linearity (19). Results from ddPCR showed good correlation with those from RT-qPCR. Due to the increased sensitivity of ddPCR, a considerable number of samples was detected by ddPCR alone. In addition, a patient's MR could be overestimated by RT-qPCR, mainly due to lower *ABL1* copies in post-TKI samples. Patients commonly experienced adverse events after TKI treatment including hematological toxicity such as anemia, leukopenia, neutropenia, and thrombocytopenia (20). Leukopenia influenced *BCR-ABL1* measurements because samples with leukopenia did not achieve enough *ABL1* copies of ≥ 10,000 for DMR and ≥ 32,000 for MR4.5. This phenomenon occurred more commonly in RT-qPCR rather than ddPCR because the latter used less sample and was reported after obtaining enough *ABL1* copies. Therefore, it seemed that ddPCR was more appropriate to monitor CML patients with leukopenia.

We also monitored 7 pediatric CML patients before and after TKI discontinuation and found that both RT-qPCR and ddPCR were useful. Except for two patients who restarted TKI due to loss of MMR, the other five maintained stable MR (better than MMR) under careful observation. One patient that suffered growth retardation has been catching up after TKI discontinuation. In addition, a condensed follow-up seemed to be necessary in pediatric CML patients after TKI discontinuation, which enabled us to evaluate patient status and to determine who needed to restart TKI at the right time. When compared the results in patients who maintained TKI more than 48 months, ddPCR revealed worse MR compared with RT-qPCR and provided more accurate information for TKI maintenance. We also found that RT-qPCR did not match the need for adequate *ABL1* copies for MR4.5. A recent study suggested that RT-qPCR was not able to discriminate patients with a higher risk of MR loss after discontinuation, whereas digital PCR suggested that the amount of *BCR-ABL1* transcript was stable (21, 22). Therefore, ddPCR was technically more acceptable to decide and monitor pediatric CML patients before and after TKI discontinuation. To our knowledge, this is the first report demonstrating the utility of ddPCR in pediatric CML patients after TKI treatment. Of course, the recommendation cannot come from observations in this study but should be further validated via more studies including a prospective design and a large number of cases.

## Data availability statement

The original contributions presented in the study are included in the article/supplementary materials, further inquiries can be directed to the corresponding author/s.

## Ethics statement

This study was approved the Institutional Review Board (IRB)/Ethics Committee of the Catholic Medical Center, Seoul, Korea (IRB No. KC19SESI0235). It was conducted in accordance with the ethical guidelines and with the 1964 Declaration of Helsinki and its later amendments or comparable ethical standards.

## Author contributions

SK, JWL, BC, N-GC, and MK collected the clinical samples and reviewed the cases. YeK performed the experiments. YeK, JML, AA, JY, YoK, and MK interpreted the results and analyzed the data statistically. YeK, SK, N-GC, and MK wrote manuscript. The authors read and approved the final manuscript. All authors contributed to the study conception and design.

## Funding

This study was supported by Research Fund of Seoul St. Mary's Hospital, The Catholic University of Korea.

## Conflict of Interest

The authors declare that the research was conducted in the absence of any commercial or financial relationships that could be construed as a potential conflict of interest.

## Publisher's note

All claims expressed in this article are solely those of the authors and do not necessarily represent those of their affiliated organizations, or those of the publisher, the editors and the reviewers. Any product that may be evaluated in this article, or claim that may be made by its manufacturer, is not guaranteed or endorsed by the publisher.

## References

[1] Millot F Baruchel A Guilhot J Petit A Leblanc T Bertrand Y. Imatinib is effective in children with previously untreated chronic myelogenous leukemia in early chronic phase: results of the french national phase IV trial. J Clin Oncol. (2011) 29:2827–32. 10.1200/JCO.2010.32.7114 21670449

[2] Pivot X Bondarenko I Nowecki Z Dvorkin M Trishkina E Ahn J-H. Phase III, randomized, double-blind study comparing the efficacy, safety, and immunogenicity of SB3 (Trastuzumab Biosimilar) and reference trastuzumab in patients treated with neoadjuvant therapy for human epidermal growth factor receptor 2–positive early breast cancer. J Clin Oncol. (2018) 36:968–74. 10.1200/JCO.2017.74.0126 29373094

[3] Hijiya N Suttorp M. How i treat chronic myeloid leukemia in children and adolescents. Blood. (2019) 133:2374–84. 10.1182/blood.2018882233 30917954

[4] Millot F Guilhot J Baruchel A Petit A Leblanc T Bertrand Y. Growth deceleration in children treated with imatinib for chronic myeloid leukaemia. Eur J Cancer. (2014) 50:3206–11. 10.1016/j.ejca.2014.10.007 25459396

[5] Samis J Lee P Zimmerman D Arceci RJ Suttorp M Hijiya N. Recognizing endocrinopathies associated with tyrosine kinase inhibitor therapy in children with chronic myelogenous leukemia. Pediatr Blood Cancer. (2016) 63:1332–8. 10.1002/pbc.26028 27100618

[6] Giona F Saglio G Moleti ML Piciocchi A Rea M Nanni M. Treatment-free remission after imatinib discontinuation is possible in paediatric patients with chronic myeloid leukaemia. Br J Haematol. (2015) 168:305–8. 10.1111/bjh.13103 25160793

[7] Saussele S Richter J Guilhot J Gruber FX Hjorth-Hansen H Almeida A. Discontinuation of Tyrosine Kinase Inhibitor Therapy in Chronic Myeloid Leukaemia (EURO-SKI): a prespecified interim analysis of a prospective, multicentre, non-randomised, trial. Lancet Oncol. (2018) 19:747–57. 10.1016/S1470-2045(18)30192-X 29735299

[8] Saußele S Richter J Hochhaus A Mahon FX. The concept of treatment-free remission in chronic myeloid leukemia. Leukemia. (2016) 30:1638–47. 10.1038/leu.2016.115 27133824 PMC4980559

[9] Deininger MW Shah NP Altman JK Berman E Bhatia R Bhatnagar B. Chronic myeloid leukemia, version 2.2021, NCCN clinical practice guidelines in oncology. J Natl Compr Canc Netw. (2020) 18:1385–415. 10.6004/jnccn.2020.0047 33022644

[10] Hochhaus A Masszi T Giles FJ Radich JP Ross DM Gómez Casares MT. Treatment-free remission following frontline nilotinib in patients with chronic myeloid leukemia in chronic phase: results from the enestfreedom study. Leukemia. (2017) 31:1525–31. 10.1038/leu.2017.63 28218239 PMC5508077

[11] Millot F Suttorp M Ragot S Leverger G Dalle J-H Thomas C. Discontinuation of imatinib in children with chronic myeloid leukemia: a study from the international registry of childhood cml. Cancers. (2021) 13:4102–10. 10.3390/cancers13164102 34439257 PMC8392145

[12] Alikian M Whale AS Akiki S Piechocki K Torrado C Myint T. RT-aPCR and RT-digital PCR: a comparison of different platforms for the evaluation of residual disease in chronic myeloid leukemia. Clin Chem. (2017) 63:525–31. 10.1373/clinchem.2016.262824 27979961

[13] Franke GN Maier J Wildenberger K Cross M Giles FJ Müller MC. Comparison of real-time quantitative PCR and digital droplet PCR for *BCR-ABL1* monitoring in patients with chronic myeloid leukemia. J Mol Diagn. (2020) 22:81–9. 10.1016/j.jmoldx.2019.08.007 31669230

[14] Scott S Cartwright A Francis S Whitby L Sanzone AP Mulder A. Assessment of droplet digital polymerase chain reaction for measuring *BCR-ABL1* in chronic myeloid leukaemia in an international interlaboratory study. Br J Haematol. (2021) 194:53–60. 10.1111/bjh.17521 34114218

[15] Hijiya N Schultz KR Metzler M Millot F Suttorp M. Pediatric chronic myeloid leukemia is a unique disease that requires a different approach. Blood. (2016) 127:392–9. 10.1182/blood-2015-06-648667 26511135 PMC4915793

[16] Swerdlow SH Campo E Harris NL Jaffe ES Pileri SA Stein H. Who Classification of Tumours of Haematopoietic and Lymphoid Tissues. 4th ed. Lyon, France: International Agency for Research on Cancer (2017). 30. p.

[17] Hughes T Deininger M Hochhaus A Branford S Radich J Kaeda J. Monitoring CML patients responding to treatment with tyrosine kinase inhibitors: review and recommendations for harmonizing current methodology for detecting *BCR-ABL* transcripts and kinase domain mutations and for expressing results. Blood. (2006) 108:28–37. 10.1182/blood-2006-01-0092 16522812 PMC1895821

[18] Cross NC. Standardisation of molecular monitoring for chronic myeloid leukaemia. Best Pract Res Clin Haematol. (2009) 22:355–65. 10.1016/j.beha.2009.04.001 19959086

[19] Chung HJ Hur M Yoon S Hwang K Lim HS Kim H. Performance evaluation of the QXDx *BCR-ABL* %is droplet digital PCR assay. Ann Lab Med. (2020) 40:72–5. 10.3343/alm.2020.40.1.72 31432643 PMC6713652

[20] Fachi MM Tonin FS Leonart LP Rotta I Fernandez-Llimos F Pontarolo R. Haematological adverse events associated with tyrosine kinase inhibitors in chronic myeloid leukaemia: a network meta-analysis. Br J Clin Pharmacol. (2019) 85:2280–91. 10.1111/bcp.13933 30907446 PMC6783623

[21] Bernardi S Malagola M Zanaglio C Polverelli N Dereli Eke E D'Adda M. Digital PCR improves the quantitation of DMR and the selection of CML candidates to TKIs discontinuation. Cancer Med. (2019) 8:2041–55. 10.1002/cam4.2087 30950237 PMC6536984

[22] Zanaglio C Bernardi S Gandolfi L Farina M Re F Polverelli N. RT-qPCR versus digital PCR: how do they impact differently on clinical management of chronic myeloid leukemia patients? Case Rep Oncol. (2020) 13:1263–9. 10.1159/000510440 33250741 PMC7670369

